# NAD-Driven Sirtuin Activation by *Cordyceps sinensis* Extract: Exploring the Adaptogenic Potential to Promote Skin Longevity

**DOI:** 10.3390/ijms25084282

**Published:** 2024-04-12

**Authors:** Ritamaria Di Lorenzo, Danila Falanga, Lucia Ricci, Antonio Colantuono, Giovanni Greco, Maura Angelillo, Fiorella Nugnes, Teresa Di Serio, Dorothea Costa, Annalisa Tito, Sonia Laneri

**Affiliations:** 1 Department of Pharmacy, University of Naples Federico II, Via Domenico Montesano 49, 80131 Naples, Italy; ritamaria.dilorenzo@unina.it (R.D.L.); teresa.diserio@unina.it (T.D.S.); sonia.laneri@unina.it (S.L.); 2 Arterra Bioscience SpA, Via Benedetto Brin 69, 80142 Naples, Italy; danila@arterrabio.it (D.F.); antonio@arterrabio.it (A.C.); fiorella@arterrabio.it (F.N.); annalisa@arterrabio.it (A.T.); 3 Vitalab Srl, Via Benedetto Brin 69, 80142 Naples, Italy; mauraangelillo@vitalabactive.com; 4 Virtus Mushroom, Via Mantini 6a, 16167 Genova, Italy; costa.dory@libero.it

**Keywords:** skin energy, cordycepin, longevity, sirtuins, adaptogen

## Abstract

In recent years, there has been increasing interest in utilizing Traditional Chinese Medicine principles and natural bioactive compounds to combat age-related ailments and enhance longevity. A *Cordyceps sinensis* mycelium hydroethanolic extract (CsEx), which was standardized in cordycepin and adenosine using UHPLC-DAD, was investigated for its adaptogenic properties using in vitro assays and a double-blind, placebo-controlled clinical trial involving 40 subjects. The CsEx demonstrated activity at a concentration of 0.0006%, significantly increasing sirtuin expression (SirT1: +33%, SirT3: +10%, SirT6: +72%, vs. CTR, *p* < 0.05) and NAD+ synthesis in HaCat cells (+20% vs. CTR, *p* < 0.001). Moreover, the CsEx boosted ATP production by 68% in skin cells, correlating with higher skin energy values (+52.0% at D_28_, *p* < 0.01) in the clinical trial. Additionally, CsEx notably reduced cytosolic reactive oxygen species (ROS) by 30% in HaCaT cells (*p* < 0.05) and enhanced collagen production both in vitro (+69% vs. CTR, *p* < 0.01) and in vivo (+10% vs. D0, *p* < 0.01), confirmed by ultrasound examination. Furthermore, CsEx’s stimulation of fibroblasts, coupled with its antioxidant and energizing properties, led to a significant reduction in wrinkles by 28.0% (D28, *p* < 0.001). This study underscores *Cordyceps sinensis* hydroethanolic extract’s potential in regulating skin cell energy metabolism and positively influencing the mechanisms associated with skin longevity control.

## 1. Introduction

The process of aging is a multifaceted phenomenon that encompasses a myriad of physiological, psychological, and biochemical changes, which occur in living organisms over time. As individuals age, they experience a gradual decline in various bodily functions, leading to an increased susceptibility to age-related disorders and a decline in their overall quality of life [1]. Despite significant advancements in medical science, the complexities of aging remain a formidable challenge, necessitating a comprehensive approach to mitigate its effects.

Traditional Chinese Medicine (TCM) offers a holistic framework that views aging as a natural process influenced by the balance and harmony of bodily systems [2]. With its roots tracing back thousands of years, TCM emphasizes the interconnectedness of mind, body, and spirit in maintaining health and well-being [3].

In recent years, there has been growing interest in leveraging the principles of TCM and the therapeutic potential of natural bioactive molecules to address age-related disorders and promote healthy aging. Natural bioactive molecules, derived from plants, herbs, and other natural sources, possess diverse pharmacological properties that can modulate the cellular processes implicated in aging [4,5,6,7], such as oxidative stress [8,9], inflammation [10,11], and cellular senescence [12,13,14].

TCM has long relied on natural products derived from plants, animals, and minerals to promote health and treat various ailments. Among these natural resources, fungi have emerged as particularly significant due to their potent therapeutic properties, especially as adaptogens [15]. Adaptogens are substances that help the body adapt to stressors and enhance their performance [16,17,18] by promoting balance and resilience without causing significant side effects. Fungi, like *Ganoderma lucidum* (Lingzhi), *Cordyceps sinensis* (Dong Chong Xia Cao), *Lentinula edodes* (Shiitake), *Auricularia auricula-judae* (Wood Ear), *Trametes versicolor* (Yunzhi), and *Tremella fuciformis* (Silver Ear), with their high contents of bioactive compounds, such as polysaccharides [19], triterpenoids [20], flavonoids and phenolic compounds [21], have been utilized in TCM for centuries to bolster the body’s defenses, enhance vitality, and restore equilibrium, with beneficial effects on the aging process by supporting longevity and vitality [22,23].

Among these, *Cordyceps sinensis* is a kind of ascomycetes parasitic fungus which belongs to the family *Clavicipitaceae* [24]. More precisely, *Cordyceps* is a genus of fungi that includes over 400 different species, including *C. sinensis*, *C. militaris*, and *C. ophioglossoides*, with different properties and potential health benefits [25].

In TCM, *Cordyceps sinensis* is highly valued for its adaptogenic properties. Prior studies have demonstrated its potential to improve mitochondrial function [26] and enhance energy metabolism while increasing oxygen utilization in cells [27], which may contribute to its anti-aging effects [28]. It is found only in the mountainous regions of China, Nepal, Bhutan, and Tibet at an altitude ranging from 3000 to 5000 m [24].

Additionally, *C. sinensis* contains bioactive compounds such as polysaccharides, cordycepin, adenosine, and many other phytochemicals that contribute to its pharmacological activity [24,26,28,29]. While *C. sinensis* has been traditionally used in TCM for various health purposes, including promoting longevity and vitality, research specifically focused on its effects on skin aging in humans is still relatively scarce and only ascribable to its photoprotection activity against UV rays [30,31]. This paper explores the potential of natural bioactive molecules in the management of age-related disorders by integrating the ancient wisdom of TCM with modern scientific advancements. More precisely, under the guidance of members of the Traditional Chinese Medicine group at the University of Florence, we prepared an extract from Cordyceps sinensis mycelium (CsEx) containing well-known TCM bioactive molecules. To explore the potential of the CsEx, its activity on several age-related markers was investigated using in vitro assays. Specifically, to determine its ability to boost longevity markers like SirT1, SirT3, and SirT6, ATP and Pro-Collagen I production were investigated along with the stress shielding effect, which was evaluated through TLR3 expression, cytosolic ROS consumption, and NAD restoration. SirT1, SirT3, and SirT6 are proteins known as sirtuins and are implicated in regulating cellular processes and extending the lifespan in various organisms. These proteins exhibit anti-inflammatory and antioxidant properties, contributing to cellular health and resilience against age-related damage [32]. Adenosine triphosphate (ATP) serves as the cellular energy currency and is crucial for sustaining cellular functions and overall health. Optimal ATP levels are associated with longevity, reflecting efficient cellular energy metabolism [33]. Pro-Collagen I, a precursor to collagen, maintains skin integrity and hydration, both crucial for a youthful appearance and overall vitality [34]. Together, these markers signify key aspects of cellular health and aging processes, highlighting their importance in longevity research and anti-aging interventions.

In this study, a *Cordyceps sinensis* mycelium hydroethanolic extract (CsEx), which was standardized in cordycepin and adenosine using the UHPLC-DAD method, was investigated for its adaptogenic molecular mechanism using in vitro assays on skin cells and a double-blind, placebo-controlled clinical trial involving 40 subjects with various skin aging-related disorders, such as a lack of firmness and laxity, wrinkles, and tired-looking and dull skin. These subjects were treated for 28 days with a topical formulation containing the CsEx, following the verification of its skin tolerability through an occlusive patch test. This comprehensive approach aims to elucidate the molecular mechanisms underlying the adaptogenic properties of the CsEx and assess its efficacy in alleviating common signs of skin aging.

The study represents a significant advance in anti-aging interventions, merging Traditional Chinese Medicine with modern science. By investigating a *Cordyceps sinensis* mycelium extract (CsEx) for its potential in managing age-related disorders, the research pioneers the exploration of the relationship between longevity markers and anti-aging effects. It also reveals the correlation between mitochondrial ATP production and skin energy for the first time, offering new insights into skin aging mechanisms. This research lays the groundwork for the development of targeted anti-aging strategies informed by both traditional knowledge and contemporary scientific understanding. Moreover, the findings validated by the clinical trial demonstrate the efficacy of *Cordyceps sinensis* in addressing skin aging concerns by promoting healthy aging through the exploitation of its adaptogenic properties.

## 2. Results

### 2.1. C. sinensis Extract Preparation and Chemical Characterization

As known, *C. sinensis* is rich in many active components, mainly including cordycepin, adenosine, sterols, and many other polysaccharides [24,29,35,36]. Cordycepin (3′-deoxyadenosine) is a nucleoside analogue composed of a purine molecule linked to a ribose sugar, and it is the most representative bioactive constituent and marker of quality in *C. sinensis* fruiting bodies. However, feeding with specific growth supplements can also enhance the production of bioactive metabolites like cordycepin in mycelial cultures of *C. sinensis* [37]. It was found that the cordycepin extraction yield increased with the effect of ultrasonication and with the use of aqueous extractive solvents with an ethanol concentration of 50% [38,39].

**Figure 1:**
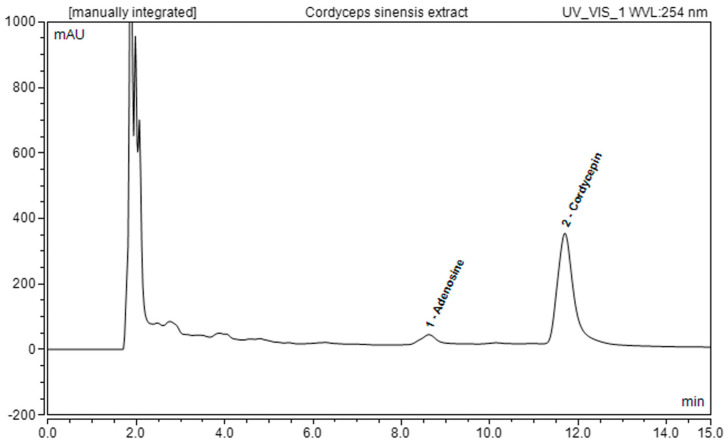
UHPLC-DAD chromatograms at 254 nm for freeze-dried *C. sinensis* hydroethanolic extract.

Specifically, cordycepin was identified as the main component of the extract, with a concentration of 6247 ± 20 µg/g of freeze-dried sample. Conversely, the adenosine content was equal to 470 ± 55 µg/g of freeze-dried sample. Figure 1 shows the chromatograms attained for the freeze-dried *C. sinensis* hydroethanolic extract from the UHPLC-DAD analysis (Table 1).

Moreover, as reported in Table 2, a comprehensive analysis of the bioactive metabolites present in the CsEx was performed to highlight other components that influenced the test results. Specifically, total polyphenol, protein, and peptide concentrations, respectively, of 21.2 ± 0.4, 23.6 ± 0.1, and 9.4 ± 0.4 mg per gram of freeze-dried sample, were calculated. Furthermore, the CsEx demonstrated a rich sugar content, with glucose, fructose, and sucrose as the main ingredients, for which the concentrations stood at 15.9 ± 0.8, 4.7 ± 0.2, and 1.4 ± 0.1 mg per gram of freeze-dried extract, respectively.

### 2.2. Biological Activity Determination

#### 2.2.1. Effect of CsEx on Cellular Longevity

Sirtuins, also called ‘longevity proteins’, are keenly involved in the life cycles of cells [32]. They are NAD+-dependent histone deacetylases, which prevent cellular senescence by engaging in DNA repair, preventing telomere attrition, and maintaining genome integrity.

**Figure 2:**
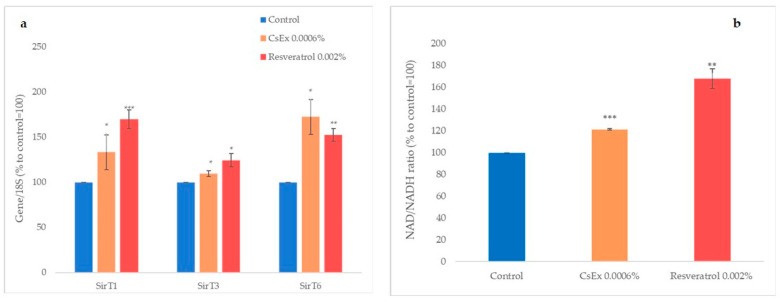
(**a**) Effect of the *C. sinensis* extract (CsEx) on the expressions of Sirtuins 1, 3, and 6 in keratinocytes. The keratinocytes were stimulated with 0.0006% CsEx or with resveratrol as a positive control for 6 h and then processed for RT-PCR analysis. (**b**) The NAD/NADH ratio was determined in HaCaT cells after 24 h of treatment with CsEx or resveratrol. The reported values represent the averages of three independent experiments; the control was set to 100%. The bars represent the standard deviations, and the asterisks indicate the *p*-value according to Student’s *t* test (* *p* < 0.05, ** *p* < 0.01, *** *p* < 0.001).

Longevity-based skin cell health was studied by monitoring SirT1, SirT3, and SirT6 expression using semiquantitative PCR, which was performed on HaCaT cells treated for 6 h with 0.0006% *w*/*v* of CsEx and resveratrol, which was used as a positive control [40]. As shown in Figure 2a, 0.0006% CsEx increased the expressions of SirT1, SirT3, and SirT6 by about 33%, 10%, and 72%, respectively. To verify if CsEx had affected the induction of NAD, the quantity of NAD in HaCaT cells treated with CsEx or with resveratrol as the positive control was measured using a specific assay. As shown in Figure 2b, the CsEx increased the synthesis of NAD by 20%, and the positive control resveratrol increased NAD by more than 50%.

#### 2.2.2. Effect of CsEx on ATP Production In Vitro and Boosting Skin Energy In Vivo

**Figure 3:**
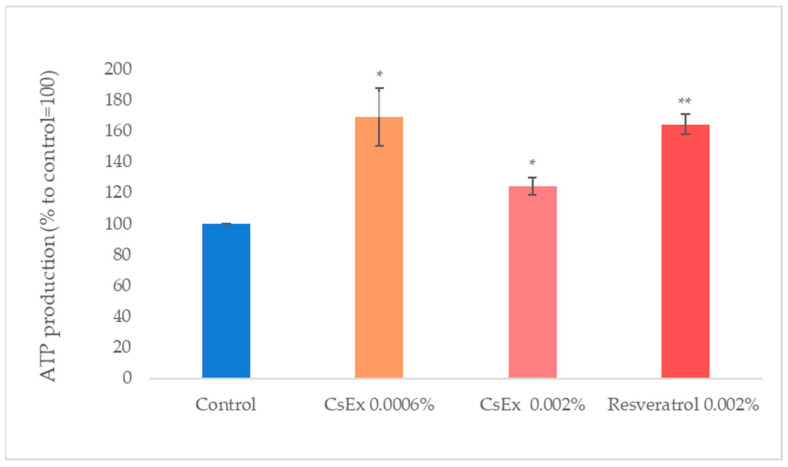
Effect of the *C. sinensis* extract (CsEx) on ATP production in keratinocytes. The keratinocytes were stimulated with 0.0006% *w*/*v* and 0.002% *w*/*v* of CsEx for 24 h and then induced to lysis. The reported values represent the averages of three independent experiments; the control was set to 100%. The bars represent the standard deviations, and the asterisks indicate the *p*-value according to Student’s *t* test (* *p* < 0.05, ** *p* < 0.01).

Mitochondria are vital organelles governing cell metabolism [41]. The quintessential role of mitochondria is the generation of ATP, adenosine triphosphate (ATP), the source of energy for most cellular processes. We measured the production of ATP in keratinocytes treated with CsEx. As shown in Figure 3, the CsEx stimulated ATP production by about 68% and 25% (at the concentrations of 0.0006% *w*/*v* and 0.002% *w*/*v*, respectively), and with a comparable efficiency to that of resveratrol.

Skin cells, like all cells in the body, undergo cellular respiration to generate energy for their metabolic processes. As a result, they do release some heat as a byproduct of this process [42].

**Figure 4:**
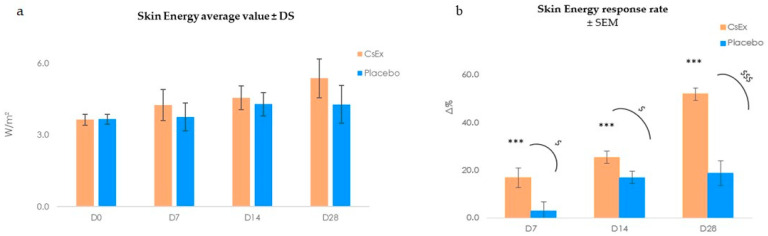
Effect of the *C. sinensis* extract (CsEx) on skin energy boosting in treated volunteers vs. placebo. (**a**) Skin energy average value ± SD; (**b**) Skin energy average percentage variation vs. D_0_. The asterisks indicate statistically significant values vs. D_0_ (*** *p*-value was between 0.0001 and 0.001). The ^$^ indicates statistically significant values vs. placebo (^$^ *p* < 0.05, ^$$$^ *p* < 0.001).

To measure heat loss in volunteers treated with the CsEx formulation, we used the Tewameter TM Hex (Courage + Khazaka electronic GmbH, Koln, Germany), a new device with 30 sensors that further measure TEWL and detect other parameters like heat loss, which is expressed in W/m^2^. This parameter is related to the local skin energy balance, which is directly related to cellular respiration, and thus, to ATP production. During the clinical trial, the skin energy was recorded after 7, 14, and 28 days of treatment with a topical cosmetic formulation containing 6 mg/L of CsEx, obtained after the dissolution of 22 mL *Cordyceps sinensis* hydroethanolic extract in 75 mL of glycerin and 3 mL of 1,2-hexanediol, thus corresponding to a final CsEx concentration of 0.0006% *w*/*v*, the same used in the in vitro assays. The results reported in Figure 4 (Tables S2 and S3) show that CsEx significantly increased skin energy during the entire treatment period (17% at D_7_; 25.5% at D_14_, both *p* < 0.001), leading up to 52.0% at the end of the study (D_28_, *p* < 0.01). Thus, the CsEx exhibited good efficacy in stimulating skin metabolism, leading to more energized skin, while the placebo showed no significant action as a skin energizer.

#### 2.2.3. Effect of CsEx on Antioxidant Activity

Antioxidant complexes and the endogenous immune systems are the skin’s front lines of defense against oxidative damage and external aggressors [7,43]. The ability to protect the skin from oxidative reactions was determined by measuring the quantity of reactive oxygen species (ROS) in HaCaT cells incubated with CsEx or ascorbic acid and then stressed with H_2_O_2_.

**Figure 5:**
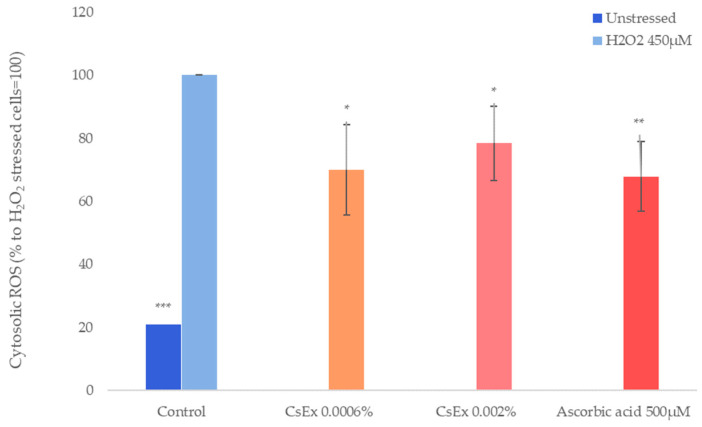
Effect of the *C. sinensis* extract (CsEx) on cytosolic ROS production in keratinocytes. The keratinocytes were stressed with H_2_O_2_ (450 µM), then treated with 0.0006% and 0.002% CsEx for 2 h and then induced to lysis. The bars represent the standard deviations, and the asterisks indicate the *p*-value according to Student’s *t* test (* *p* < 0.05, ** *p* < 0.01, *** *p* < 0.001).

As shown in Figure 5, cytosolic ROS production was significantly reduced by the CsEx treatment (30% and 22% at concentrations of 0.0006% *w*/*v* and 0.002% *w*/*v*, respectively), as well as by ascorbic acid, which was used as positive control.

#### 2.2.4. Effect of CsEx on Collagen Production

According to CsEx’s action on cell metabolism and oxidative protection, in order to analyze its role in collagen induction, we measured newly synthesized collagen type I in human dermal fibroblasts (HDFs) treated with CsEx or with TGFβ as the positive control.

**Figure 6:**
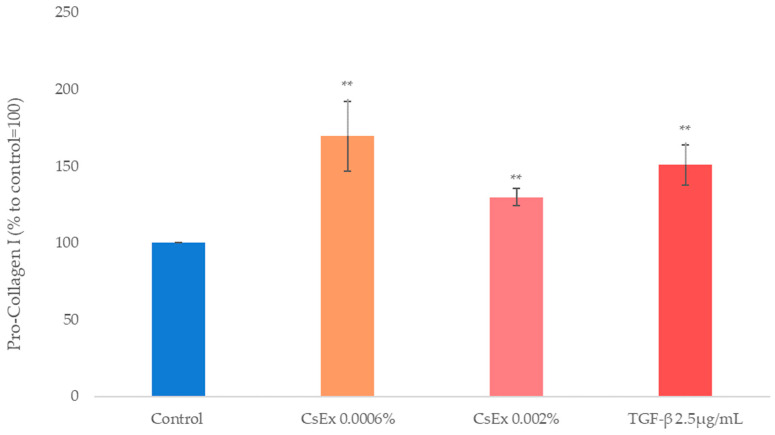
Effect of the *C. sinensis* extract (CsEx) on Pro-Collagen I production in skin fibroblasts. The cells were stimulated with 0.0006% and 0.002% CsEx for 24 h. The reported values represent the averages of three independent experiments. The asterisks indicate statistically significant values (** *p*-value was between 0.001 and 0.01).

As shown in Figure 6, CsEx at both concentrations significantly stimulated Pro-Collagen I production, analogously to the positive control TGFβ, suggesting a positive role of the extract in maintaining a correct dermis tone (Figure 5).

**Figure 7:**
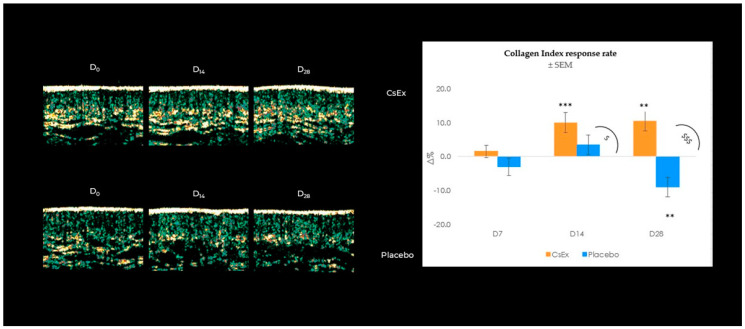
Effect of the *C. sinensis* extract (CsEx) on collagen production during 28-day topical application. Collagen index average percentage variation vs. D_0_ recorded for 20 volunteers throughout the study period (after 7, 14, and 28 days of treatment) for CsEx and placebo, intra-group difference *t*-test ** *p* < 0.01, *** *p* < 0.001), and inter-group difference ANOVA test ^$^ *p* < 0.05, ^$$$^ *p* < 0.001). The echo-graphic images were obtained with a 20 MHz HFUS ultrasound probe (DermaScan^®^ C, Cortex Technology Aps, Aalborg, Denmark), after placing it on test subjects’ faces.

To confirm the promising in vitro results on boosting collagen in fibroblasts, the ability of the formulated CsEx to increase collagen production in enrolled subjects was investigated. Collagen levels were checked using ultrasound detection at all follow-ups, and as indicated in Figure 5, CsEx was able to significantly induce collagen after 14 and 28 days by 10.5 and 10.0% (Table S4) compared to the placebo (Table S5), which showed no action on collagen production. Still, as shown in the echo-graphic images reported in Figure 7, CsEx densified the skin by increasing the collagen amount and improving the dermis bundles.

#### 2.2.5. Effect of CsEx on Wrinkle Appearance Reduction

**Figure 8:**
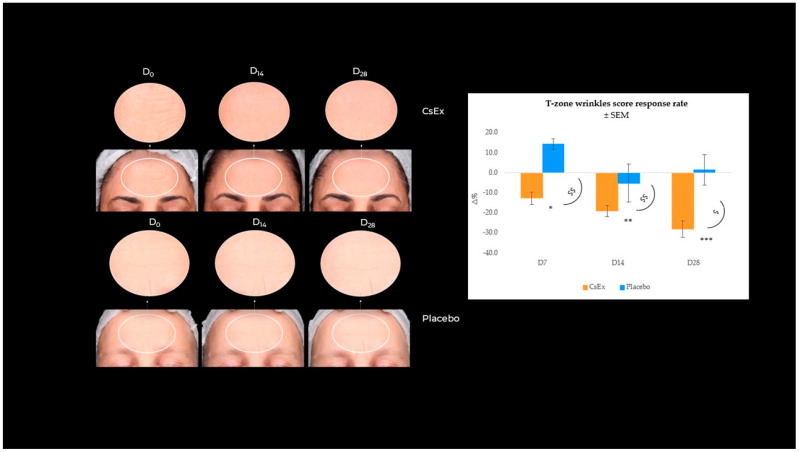
Effect of the *C. sinensis* extract (CsEx) on collagen production during 28-day topical application. Collagen index average percentage variation vs. D_0_ recorded for 20 volunteers throughout the study period (after 7, 14, and 28 days of treatment) for CsEx and placebo, intra-group difference *t*-test * *p* < 0.05, ** *p* < 0.01, *** *p* < 0.001), and inter-group difference ANOVA test ^$^ *p* < 0.05, ^$$^ *p* < 0.01). The images of forehead wrinkles over a 28-day treatment with 6 mg/L CsEx vs. placebo were obtained with a Visia 7th (Canfield Scientific Inc., Parsippany, NJ, USA).

The aging process alters both the structure and mechanical properties of the skin; as a result, the skin gets more wrinkles [44]. Microscopically, the fine mesh of the skin surface declines, and each wrinkle becomes prominent, as its width and height grow with age [44]. Thus, the demonstrated stimulating action of the CsEx on fibroblasts along with its antioxidant and energizing activity, with reasonable predictivity, has a pivotal role in the phenotypic manifestations of skin aging, like wrinkles and fine line appearance. As evidenced, our study demonstrated that the CsEx treatment decreased the most evident facial wrinkles. Specifically, T-zone wrinkles (forehead and frown lines) were detected using a VISIA 7th (Canfield Scientific Inc., Parsippany, NJ, USA), which showed a statistically significant decrease over time by −12.5, −19.0, and −28.0% at D_7_, D_14_, and D_28_ vs. baseline (Figure 8), contrary to what was observed for the placebo-treated group (Tables S6 and S7). These results demonstrated the CsEx’s validity in the treatment of the most typical skin aging phenomena.

## 3. Discussion

The study delves into the multifaceted processes of aging, particularly focusing on skin aging, which is heavily influenced by both intrinsic and extrinsic factors. Aging is an inevitable process based on a gradual decline, with radical changes in many processes of the body [45].

Central to this investigation are sirtuins, which are essential proteins implicated in cellular processes crucial for longevity and antioxidant protection [32,46,47]. Sirtuins, a family of NAD+-dependent histone deacetylase, are involved in processes like glucose and lipid metabolism [48], mitochondrial biogenesis [49], DNA repair [50], oxidative stress, apoptosis, and inflammation [51]. For this reason, sirtuins are considered key proteins in longevity control. Different strategies are already used to increase their expression and their activity to improve longevity and guarantee better antioxidant protection and improved metabolism; one of these is the employment of phytochemicals, which are easily accessible compounds. The research explores the potential of *Cordyceps sinensis* mycelium extract (CsEx) in managing age-related disorders by targeting sirtuin expression, through its incorporation in a topical formulation. *C. sinensis* has a long history of use in Traditional Chinese Medicine, where it has been valued for its apparent adaptogenic properties. Recently, there has been great attention on the individuation of natural compounds with adaptogen activities in order to enhance the body’s resilience to stress and support overall vitality and endurance. Specifically, it was well-established that *Cordycepin*, the characteristic molecule of this mushroom, is a natural compound with multi-target cosmeceutical potential. In particular, the contents of cordycepin and adenosine are markers of the quality of *C. sinensis* fruiting bodies, and feeding with specific growth supplements can enhance the production of these bioactive metabolites, even in the mycelium [34]. The CsEx employed in this study was standardized in order to reach a cordycepin concentration of 6247 ± 20 µg/g and an adenosine content of 470 ± 55 µg/g.

Recently, the importance of inducing not only the expression of sirtuins but also their availability in a way to have a real effect on sirtuin enzymatic activity was reported [47]. Through both in vitro and in vivo assays, the CsEx demonstrated its ability to enhance sirtuin expression (SirT1: +33% vs. CTR, *p* < 0.05; SirT3: +9% vs. CTR, *p* < 0.05; SirT6: +72% vs. CTR, *p* < 0.001) in cultured keratinocytes, which is crucial for cellular energy metabolism and parallelly to induce NAD+ synthesis (+20% vs. CTR, *p* < 0.001), giving a double effort in sirtuin activation.

By examining the pivotal role of NAD+ in the regulation of cellular energy metabolism [52], the study further reveals CsEx’s remarkable impact on ATP synthesis, which is indicative of its potent influence on cellular energy dynamics, a factor vital for skin health and longevity. Through an in vitro test conducted on keratinocytes, we observed a substantial increase in ATP production compared to untreated cells (+68% vs. CTR), underscoring the potent influence of the CsEx on cellular energy dynamics. This effect was confirmed during the instrumental clinical trial performed on healthy human subjects who were treated for 28 days with the CsEx-based topical formulation (6 mg/L) by using the innovative system Tewameter TM Hex (Courage+Khazaka electronic GmbH, Köln, Germany), a device capable of measuring heat loss, the parameter intricately linked to the local skin energy balance.

Remarkably, individuals treated with the CsEx exhibited a significant increase in this parameter, providing concrete evidence of the formulation’s impact on skin energy. This promising result could be mainly due to the CsEx-relevant cordycepin and adenosine standardized content. In this regard, from the molecular point of view, it was described that cordycepin can activate AMP protein kinase (AMPK), a cellular energy sensor that regulates energy metabolism [53]. Specifically, it was reported that cordycepin enters cells via adenosine transporters and is converted by cellular metabolism into mono-, di-, and triphosphates, which at high cordycepin concentrations, can almost replace cellular adenine nucleotides. This explains why cordycepin can activate AMPK, leading to increased mitochondrial biogenesis and enhanced oxidative metabolism, thereby promoting energy production by mimicking multiple effects of its natural activator, adenosine monophosphate (AMP). As known, ATP production is the final step of cellular respiration, an overall process that involves multiple steps, each of which contributes to the release of energy in the form of ATP. Cellular respiration is an exergonic process. This means it releases energy that is used by the cell for various metabolic processes, and some of this energy is released as heat [40]. This is particularly evident in endothermic organisms, such as mammals and birds, meaning they maintain a constant internal body temperature. In these organisms, cellular respiration is a major source of heat production, which helps regulate their body temperature and maintain metabolic functions. Our clinical trial corroborated these findings, showing significant improvements in the skin energy balance in subjects treated with the CsEx-based formulation (+52% vs. D_0_ after 28 days, *p* < 0.001). As another consequence of the mitochondrial activity stimulation, the CsEx improved the ability of dermal fibroblasts to synthesize new collagen. Notably, a reduction in skin wrinkles (−28.0% vs. D_0_, *p* < 0.001) and an increase in collagen (+10% vs. D_0_ after 28 days, *p* < 0.001) production were observed, affirming the CsEx’s potential as an effective anti-aging solution.

These findings illuminate novel applications of a *Cordyceps sinensis* extract in modulating energy metabolism within skin cells, thus positively influencing skin longevity control [54,55,56].

Moreover, the study underscores the importance of integrating traditional wisdom with modern scientific understanding to develop targeted anti-aging therapies with tangible clinical benefits. Finally, the comparison between the in vitro and clinical outcomes emphasizes the effectiveness of CsEx-based cosmetic formulations in translating molecular findings into tangible improvements in skin health and appearance.

## 4. Materials and Methods

### 4.1. Reagents and Human Cell Cultures

All chemicals, reagents, and standards used were analytical or LC-MS-grade reagents. The water was treated in a Milli-Q water purification system (Millipore, Bedford, Burlington, MA, USA) before use. Sunflower oil was purchased in a local market. Cordycepin (purity ≥ 95.0% HPLC) and adenosine (purity ≥ 99% HPLC) were purchased from Sigma-Aldrich (Milan, Italy). For the o/w topical formulation, cosmetic-grade ingredients were used, such as Carbomer, Sodium Gluconate, Glyceryl Stearate, PEG-100 Stearate, Caprylic/Capric Triglycerides, Cetearyl Alcohol, Cetyl Ricinoleate, Phenoxyethanol, and Ethylhexylglycerin. All listed excipients were purchased from ACEF Spa (Fiorenzuola D’arda, Italy).

Immortalized human keratinocytes (HaCaT) bought from Addexbio Technologies (San Diego, CA, USA) were preserved in Dulbecco’s Modified Eagle Medium (DMEM; Sigma Aldrich, St. Louis, MO, USA) that was supplemented with 10% fetal bovine serum (FBS; Sigma-Aldrich, St. Louis, MO, USA) in 95% air, 5% CO_2_, and a humidified atmosphere at 37 °C. Human dermal fibroblasts (HDFs) were preserved in Dulbecco’s Modified Eagle Medium (DMEM; Sigma-Aldrich, St. Louis, MO, USA) supplemented with 10% of fetal bovine serum (FBS; Sigma-Aldrich, St. Louis, MO, USA) in 95% air, 5% CO_2_, and a humidified atmosphere at 37 °C.

### 4.2. Cordyceps sinensis Preparation and Extraction of Cordycepin Fraction

*C. sinensis* mycelium was isolated by the farm Virtus Mushrooms located in Genova (Italy) from sporophores originating from Tibet, which were incubated and isolated in an agar medium containing glucose (10 g/L), maltose (5 g/L), yeast extract (1.5 g/L), peptone (1.5 g/L), MgSO_4_ (0.5 g/L), and KH_2_PO_4_ (0.5 g/L). The isolated mycelium was then propagated on a culture medium composed of ground millet, rice bran, and rice husk in specific cultivation bags at 20 °C, with controlled humidity and temperature. These conditions were maintained for 70 days in a way to guarantee the complete development and the highest synthesis of cordycepin. Finally, the mycelium was air dried at 48 °C and then pulverized.

The simultaneous extraction of adenosine and cordycepin was achieved by using an extractive protocol adopted by Thanh et al. [57]. Specifically, 2000 mL of an ethanol/water (50/50, *v*/*v*) solution was added to 20 g of dried mycelium. The mixture was homogenized for 3 min at 1500 rpm and 6 min at 3800 rpm using a Grindomix GM300 knife mill (Retsch GmbH, Haan, Germany). The resulting suspension was stirred at 400 rpm for 2 h at 25 °C, avoiding light exposure. Then, the suspension was centrifuged at 6300 rpm for 30 min at 4 °C. The supernatant was removed, filtered, and then concentrated up to 20% of the starting volume under vacuum in a rotary evaporator (IKA RV8, IKA-Werke GmbH & Co., Staufen, Germany) set to 25 °C. Finally, the pH was brought to 7.0 with 10 N NaOH, and the extract was freeze dried for the chemical characterization. The amounts of adenosine and cordycepin were quantified using a UHPLC-DAD analysis. The extraction process was performed in triplicate.

Specifically, adenosine and cordycepin extractions from freeze-dried *C. sinensis* hydroalcoholic extracts were performed by using the following procedure adapted from Huang et al. [58], where about 20–40 mg of sample was accurately weighed in a 15 mL Falcon tube. One mL of a methanol/water solution (50:50) was added to each sample. An ultrasound-assisted extraction was performed using an ultrasonic bath (37 KHz at room temperature for 30 min). At the end of the extraction process, the samples were centrifuged at 4500 rpm for 10 min at 4 °C. The supernatant was collected and further centrifuged at 17,850 rpm for 10 min at 4 °C. After passing through a Phenex-RC 0.20 µm syringe filter, the samples were injected for chromatographic analysis. Finally, the *C. sinensis* hydroethanolic extract was employed for the in vitro assays at the indicated concentrations. Then, 22 g of CsEx was incorporated into 75 g of glycerin and 3 g of 1,2–hexanediol as a preservative system to obtain the cosmetic active ingredient to be employed in the clinical trial.

### 4.3. Identification and Quantification of the Cordycepin and Adenosine

A UHPLC analysis of adenosine and cordycepin was performed using a Vanquish™ Flex UHPLC system (Thermo Fisher Scientific, Waltham, MA, USA) equipped with a VF-D11-A Diode Array Detector (DAD) set at 254 nm (Thermo Fisher Scientific, Waltham, MA, USA). The system consisted of a VF-P10-A binary pump, a VF-10A-A thermostated autosampler (4 °C), and a VC-C10-A thermostated column compartment (Thermo Fisher Scientific, Waltham, MA, USA). Chromatographic separation was achieved through a Hypersil Gold Vanquish C18 column (150 mm × 2.1 mm, 1.9 μm) equipped with a Hypersil Gold security guard and thermostated at 30 °C (Thermo Fisher Scientific, Waltham, MA, USA). The mobile phase consisted of water/methanol (92:8, *v*/*v*). The flow rate was set to 200 μL/min, and the injection volume was 2.5 μL. The data were evaluated using Chromeleon Software 7.2.10 (Thermo Fisher Scientific, Waltham, MA, USA). The calibration curves for the quantification were obtained by using authentic standards of adenosine and cordycepin. The analysis was performed in triplicate.

### 4.4. CsEx Bioactive Metabolite Content Analysis

The phenolic compounds’ contents were determined in triplicate using the Folin–Ciocalteu spectrophotometric method, described by Box et al. [59], using gallic acid as a reference standard; instead, the total protein was measured on the extract with Bradford reagent (Biorad Laboratories, Hercules, CA, USA) [60].

Sucrose, fructose, and glucose levels were measured using an enzyme assay (Megazyme Ltd., Bay, Ireland) according to Waleckx et al.’s procedure [61]. Finally, the total peptides were measured using the *o*-phtaldialdehyde assay (Sigma-Aldrich, St. Louis, MO, USA) according to Zahir et al.’s method [62].

### 4.5. Sirtuin Expression Assay

To analyze the gene expression in basal conditions, 1.2 × 10^5^ HaCaT cells were seeded in 6-well plates, grown for 20 h, and then treated with the extract (0.0006% *w*/*v*) or resveratrol (100 µM, 0.002% *w*/*v*) for 6 h. The total RNA was extracted with a Pure link RNA mini kit (Invitrogen-Thermo Scientific, Waltham, MA, USA). Semi-quantitative RT-PCR analyses were conducted using the pair of universal primers 18S primer/competimer (Invitrogen-Thermo Scientific, Waltham, MA, USA) as the internal standards. The PCR products were separated on a 1.5% agarose gel and viewed using the iBright instrument (Invitrogen-Thermo Scientific, Waltham, MA, USA). The sequences of the primers used for amplification were as follows: Sirt1-F ATACCCCATGAAGTGCCTC; Sirt1-R: CGTCATCTTCAGAGTCGTA; Sirt3-F: TTGGCCAAGGAGCTGTACC; Sirt3-3: TGGCAAAAGGCTCCACCT; Sirt6-F: ATCACGCTGGGTACATCG; and Sirt6-R: ACCTCGTCAACGTAGCCAT.

### 4.6. NAD/NADH Ratio Assay

To analyze the increase in the NAD/NADH ratio, 1.5 × 10^5^ HaCaT cells were seeded in 6-well plates and grown for 20 h. The cells were then incubated with 0.0006% *w*/*v* of CsEx or resveratrol (100 μM, 0.002% *w*/*v*), which was used as a positive control, for 24 h. The NAD/NADH ratio was determined using the NAD/NADH quantitation kit according to provider instructions (Merck KGaA, Darmstadt, Germany). The absorbance values were measured using the instrument Victor Nivo (PerkinElmer Inc., Singapore).

### 4.7. ATP Production Assay

To assess ATP production, 1.2 × 10^4^ HaCaT cells were seeded in 96-well plates and grown for 20 h. The cells were then incubated with extract (0.0006% and 0.002% *w*/*v*) or resveratrol (100 μM) for 24 h. After the treatment, the cells were incubated at room temperature for 30 min in a culture medium before adding an equal volume of CellTiter-Glo Reagent (Promega Corporation, Milano, Italy) to induce cell lysis. The luminescent signal of the samples is thus measured by the instrument Victor Nivo (PerkinElmer Inc.), after 15 min.

### 4.8. Cytosolic ROS Assay

To analyze the reduction of cytosolic ROS, 1.5 × 10^4^ HaCaT cells were seeded in 96-well plates, grown for 20 h and then incubated with extract (0.0006% and 0.002% *w*/*v*) or Ascorbic acid 500 μM, used as a positive control, for 2 h. After incubation, the cells were washed in PBS and incubated with the dye CM-DCFDA (5-6)-chloromethyl-2,7-dichloro-dihydrofluoresceine diacetate, (Invitrogen-Thermo Scientific, Waltham, MA, USA) at 37 °C for 45 min. After an additional wash in PBS, the cells were stressed with H_2_O_2_ 450 μM and incubated for 30 min. The fluorescence of the samples was thus measured at 535 nm (excitation 485 nm), using the instrument Victor Nivo (PerkinElmer Inc.).

### 4.9. Collagen Production Assay

To determine Pro-Collagen I production, 8 × 10^4^ HDF were seeded in 96-well plates and treated for 24 h with the extract or transforming growth factor beta (TGFβ; 2.5 ng/mL) as the positive control. After washing in phosphate-buffered saline (PBS; Thermo FisherScientific, Waltham, MA, USA), the cells were fixed in 4% *w*/*v* paraformaldehyde for 10 min, washed three times with ELISA buffer (PBS 1X, 0.5 mM CaCl_2_,1 mM MgCl_2_, 0.1% Triton X100), and treated with 3% BSA for 30 min in ELISA buffer. After 2 h of incubation with the primary antibody (sc-166,572, Santa Cruz Biotechnology, Dallas, TX, USA) at room temperature, the cells were washed, incubated with the anti-mouse secondary antibody (Biorad, Hercules, CA, USA) for 1 h, and then the amount of Pro-Collagen I was measured through a colorimetric reaction, using 0.35 mg/mL solution of OPD (Sigma-Aldrich, Milano, Italy) and 0.012% H_2_O_2_ in 50 mM citrate buffer [63].

### 4.10. Statistical Analysis in Pre-Clinical Studies

All the values reported were the average of three independent experiments, each of which was performed in triplicate. The asterisks indicate the statistical significance calculated in agreement with the *t*-test: * means the *p*-value is 0.05, ** means the *p*-value is 0.01, and *** means the *p*-value is 0.001.

### 4.11. CsEx-Based Topical Formulation Preparation

The cosmetic o/w emulsions were prepared with cosmetic-grade ingredients. First, phase A, containing oily constituents such as Glyceryl Stearate and PEG-100 Stearate (4.00% *w*/*w*), Caprylic/Capric Triglycerides (10.00% *w*/*w*), Cetearyl Alcohol (0.50% *w*/*w*), and Cetyl Ricinoleate (2.00% *w*/*w*), were mixed using a magnetic stirrer at 200 ± 25 rpm and heated to 70 ± 5 °C.

Once melting occurred, phase A was mixed with phase B (aqueous phase), comprising deionized water (81.30% *w*/*w*), Sodium Gluconate (0.10% *w*/*w*), and Carbomer (0.60% *w*/*w*), previously heated at the same temperature as phase A. Phase mixing occurred using the mechanical stirrer Silverson L5T Laboratory Mixer (SBL, Shanghai, China) at 5000–5500 rpm, until a temperature of 45 ± 5 °C was reached. Then, the mix was left to cool, and once a temperature of 35 ± 2 °C was reached, the final phase (phase C) containing the preservative system, such as a solution of Phenoxyethanol (and) Ethylhexylglycerin (0.90% *w*/*w*), was added to the mix. For the clinical trial, two batches of the topical formulation were prepared: the placebo and CsEx-based batch; this one was enriched with the active ingredient CsEx (6 mg/L) in phase C before neutralization with NaOH (20% sol.) up to pH 5.30.

#### Quality Control and Potting of the Cosmetic Formulations

After 24 h, the viscosity (27.197–28.076 mPa; 22 °C, spindle 64, 20 rpm) was measured using the Brookfield DV-E viscosimeter (AMETEK Inc., Berwyn, PA, USA), and the pH (5.2 ± 0.3) was checked with the GPL20 pH meter (Crison Instruments S.A., Barcelona, Spain). The placebo emulsions were prepared analogously and contained all the listed ingredients, except for the CsEx. Once ready, the cosmetic creams were placed in unlabeled 30 mL jars by a technician in the blind.

### 4.12. Clinical Study

The randomized, double-blind, 2-parallel arm efficacy study was performed at RD Cosmetics of the University of Naples Federico II Pharmacy Department, which has a quality management system for cosmetic clinical trials certified by IMQ S.p.A (Milan, Italy) according to the UNI EN ISO 9001 standard [64]. The study was run from September 2023 to November 2023 and aimed to assess the skin efficacy of the CsEx vs. placebo as an energizing and anti-wrinkle ingredient when incorporated in a topical formulation. Due to the cosmetic nature of the present study and the involvement of healthy adult subjects, a submission to the relevant ethics committee was not required [65]. The clinical study started after written informed consent was provided by all subjects. Nevertheless, the study was performed in accordance with the principles embodied by the Declaration of Helsinki [66] and following the Cosmetics Europe guidelines for the Evaluation of the Efficacy of Cosmetic Products [67]. Good clinical practice was maintained throughout the study period.

#### 4.12.1. Study Design and Participants

A total of 40 subjects aged 40 to 65 years (included), with Fitzpatrick skin types I–III and a dull face marked by aging with wrinkles and fine expression lines, were included in this study. Only generally healthy subjects were enrolled and randomly assigned to the two treatment groups and received the test sample according to the test group they belonged to:

- Group 1: topical formula containing 6 mg/L CsEx;
- Group 2: placebo.

Before participating, each subject signed a written informed consent that contained the aim and the type of the study, the list of the cosmetic-grade ingredients employed, the application rules, and any known or potential adverse reactions that might result from using the test products. At each check-up, the subjects were asked if they complied with all pre-visit instructions and restrictions to determine whether they were qualified to continue the study. Protocol deviations were recorded to determine which subjects, endpoints, and time points should be excluded from the subsequent data analysis.

Additional inclusion criteria involved discontinuing all cosmetic products intended for treating aging-related disorders from the point of initial screening until the final follow-up visit. However, cleansing preparations approved by the investigator and the use of makeup were permitted. The exclusion criteria comprised any dermatological condition, prior use of retinoids, and recent utilization of medical aesthetic procedures such as bio-stimulation, filler injections, or Botox within 12 weeks or five half-lives of screening. Furthermore, patients with face radiofrequency treatments within 8 weeks of screening or oral supplementation with anti-aging/antioxidant agents within 4 weeks of screening were excluded. Further exclusion criteria encompassed concurrent topical and systemic pathologies or diseases. The characteristics of the study populations are detailed in Table 3.

**Figure 9:**
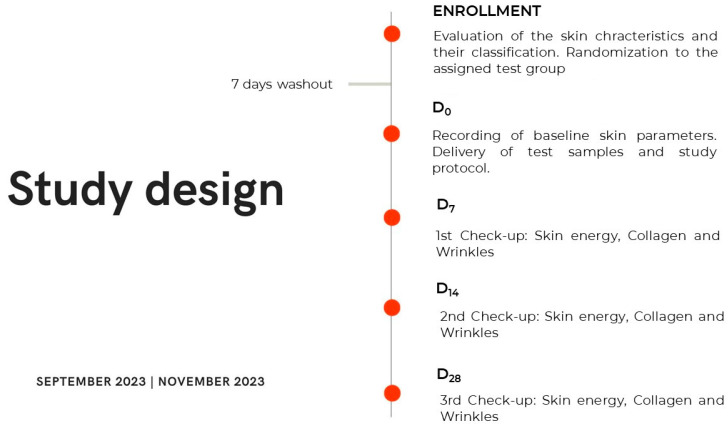
Study design scheme. D0: baseline skin condition and start of topical treatment with the assigned test sample; D7: 7 days after twice-daily test sample topical application; D14: 14 days after twice-daily test sample topical application; D28: 28 days after twice-daily test sample topical application.

Following the enrollment visit, a washout period of 7 days was mandated before the participants could enter the study. During the baseline (Day 0) and follow-up visits, the participants underwent an assessment for the monitored skin parameters and were randomly allocated to a treatment group. They were provided with test samples and instructed on their application method. The participants were required to apply approximately 2 mg of the assigned product to their face twice daily (morning and evening) for 28 days. An overview of the study design and protocol is presented in Figure 9.

The primary objective of the study was to evaluate the energizing activity of the extract once its tolerability was ensured. Secondary outcomes included an assessment of the improvements in skin aging-related parameters like skin roughness and tonicity, which were a consequence of the antioxidant and metabolism stimulation exerted by the extract.

#### 4.12.2. Randomization and Masking

The laboratory personnel utilized a system to manage study enrollment and ensure each participant’s anonymity. This system assigned each enrolled subject a unique patient study number, which was randomized based on predefined parameters established on a general panel where volunteers were recorded, while also maintaining treatment masking. Participants were drawn from diverse social backgrounds and joined the study voluntarily and prior to their enrollment, they underwent a clinical skin examination.

The system employed a customizable algorithm with defined limits for patient age (≤40 and >65 years) and skin condition (thin dermis and presence of wrinkles). Panelists were randomly allocated to receive either the CsEx-based cream (0.5% *w*/*w*) or a placebo twice daily in a 1:1 ratio. Throughout the study, panelists and investigators (excluding members of the primary endpoint analysis data monitoring teams) remained blinded to each patient’s treatment assignment. Emergency unmasking was only performed in instances of side effects necessitating the investigator’s knowledge of the subject’s treatment group.

At the conclusion of the study, it was required that 20 patients in each treatment group strictly adhered to the protocol without any significant deviations that could affect the study results.

#### 4.12.3. Local Energy Balance Assessment

Skin energy was evaluated after the repeated application of the investigated topical formulations using Tewameter^®^ TM Hex (C + K electronic GmbH, Cologne, Germany). All parameters were evaluated at baseline (D_0_) after 7, 14, and 28 days of twice-daily treatment with the assigned product (D_7_, D_14_, and D_28_).

#### 4.12.4. Anti-Aging Efficacy Assessment

Collagen production and roughness reduction were assessed after 7, 14, and 28 days (D_7_, D_14_, and D_28_) of treatment with the CsEx-based emulsion or placebo using Dermascan^®^ C (Cortex Technology Aps, Aalborg, Denmark) and VISIA 7th (Canfield Scientific Inc., Parsippany, NJ, USA), respectively.

#### 4.12.5. Skin Tolerability Test

To assess the tolerability of CsEx, its potential irritant properties were evaluated using a 48 h occlusive patch test on intact human skin, specifically on the volar forearm due to its favorable characteristics for such testing. This test aimed to identify and classify CsEx’s irritant potential in accordance with EEC Directive 76/768 [68]. Finn Chambers^®^ AQUA patch delivery system was utilized for the test, following the previously established published procedures [8]. The results (Figure S1 and Table S1) were evaluated based on the morphological criteria recommended by the International Contact Dermatitis Research Group [69], with an irritancy limit set at 1.5 on a 0–3 scale for the visual score.

### 4.13. Statistical Analysis in Clinical Trial

A sample size of 40 panelists, randomly assigned (approximately 20 subjects per group), was deemed adequate to ensure a sufficient statistical power for detecting differences between the CsEx cream and placebo. For both primary and secondary endpoints, inter-group differences were evaluated using the ANOVA test (Tables S7–S10), while intra-group differences, expressed as the average percentage variations compared to the baseline, were assessed using the Student *t*-test. The significance level for all analyses was set at an overall two-sided *p*-value of 0.05. The statistical analyses were performed using SAS software version 9.4.

## 5. Conclusions

In conclusion, the current research sheds light on the promising anti-aging prop-erties of a *Cordyceps sinensis* mycelium extract (CsEx). The study demonstrates significant increases in sirtuin expression, NAD+ synthesis, ATP production, ROS scavenging, and collagen synthesis in response to the CsEx treatment, offer exciting prospects for promoting skin longevity by exploiting natural adaptogen sources. Our future research directions can focus on advancing the understanding of the CsEx’s mechanism of action, identifying other molecular targets involved, optimizing its efficacy, and extending the duration of the relatively short clinical trial to overcome the limitations of the present study.

## Figures and Tables

**Table 1:** Adenosine and cordycepin identified and quantified in CsEx through UHPLC-DAD analysis (*n* = 3). Results are expressed as the means ± standard deviations (µg of identified compound/g freeze-dried sample).

Identified Compound	Quantified Amount in Hydroethanolic Extract (µg/g Freeze-Dried Sample)
Cordycepin	6247 ± 20
Adenosine	470 ± 55

**Table 2:** Bioactive metabolite analysis of the CsEx. Results are expressed as means ± standard deviations (mg of identified compound/g freeze-dried sample).

Sample	Total Polyphenols (mg Gallic Acid equiv./g of Freeze-Dried Extract)	Total Protein (mg BSA equiv./g of Freeze-Dried Extract)	Total Peptides (mg L-Serine equiv./g of Freeze-Dried Extract)	Glucose (mg/g of Freeze-Dried Extract)	Fructose (mg/g of Freeze-Dried Extract)	Sucrose (mg/g of Freeze-Dried Extract)
*C. sinensis* hydro-ethanolic extract	21.2 ± 0.4	23.6 ± 0.1	9.4 ± 0.4	15.9 ± 0.8	4.7 ± 0.2	1.4 ± 0.1

**Table 3:** Characteristics of study participants.

Characteristics of Study Participants	CsEx	Placebo
No. of subjects		
Female, *n* (%)	20 (100)	20 (100)
Male, *n* (%)	-	-
Mean age ± SD ^a^, years		
(min–max)	47.5 ± 6.3 (41–62)	49.1 ± 7.2 (42–65)
Fitzpatrick skin phototype, *n* (%)		
I	2 (10)	1 (5)
II	15 (75)	16 (80)
III	3 (15)	3 (25)
IV	-	-
Signs of aging, *n* (%)		
Wrinkles	20 (100)	20 (100)
Hyperpigmentation	5 (25)	3 (15)
Skin laxity	10 (50)	8 (40)
Dermis thinning	12 (60)	10 (50)

^a^ SD: standard deviation.

## Data Availability

Data supporting the findings of this study are available from the corresponding author upon reasonable request.

## Supplementary Materials

The supporting information can be downloaded at: https://www.mdpi.com/article/10.3390/ijms25084282/s1.
