# Supplementary material for: NAD-Driven Sirtuin Activation by Cordyceps sinensis Extract: Exploring the Adaptogenic Potential to Promote Skin Longevity
Source: Int J Mol Sci. 2024 Apr 12;25(8):4282. doi: 10.3390/ijms25084282 (PMC11049886; doi:10.3390/ijms25084282)
Supplement: Supplementary file 1 [file ijms-25-04282-s001.zip › ijms-2914420-supplementary 1.pdf]

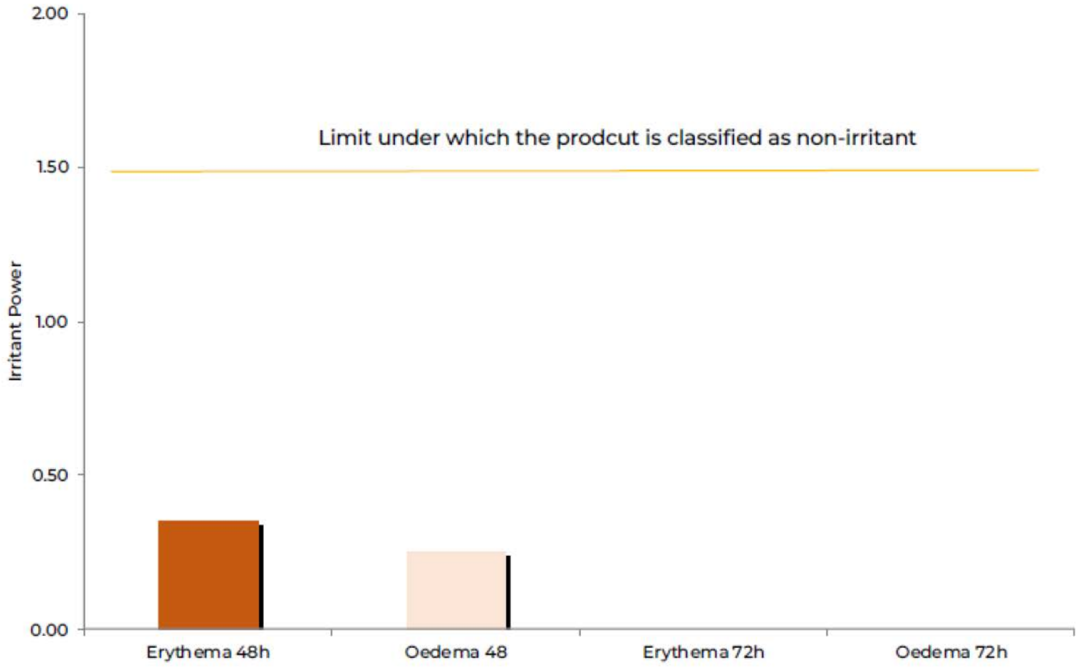

Figure S1. Value of the average irritation index.

Table S1. Value of edema and/or erythema detected after 48 and 72 hours by occlusive CsEx-containing patch application.

#	Panelist ID	Sex	Erythema 48h	Oedema 48	Erythema 72h	Oedema 72h	
1	IN97	F	1	0	0	0	
2	LR95	F	0	0	0	0	
3	CF98	F	0	0	0	0	
4	RDL92	F	0	0	0	0	
5	EV79	F	0	0	0	0	
6	SL68	F	2	0	0	0	
7	AM68	F	1	0	0	0	
8	IC99	F	0	1	0	0	
9	IN77	F	1	2	0	0	
10	VN76	F	0	0	0	0	
11	CS98	F	0	0	0	0	
12	FS77	F	1		0	0	
13	EV00	F	0	0	0	0	
14	AD66	F	0	1	0	0	
15	PR71	F	0	0	0	0	
16	RB76	F	0	0	0	0	
17	GS88	F	0	1	0	0	
18	MDC76	F	1	0	0	0	
19	RC99	F	0	0	0	0	
20	LC00	F	0	0	0	0	
MEAN VALUES			**0.350**	**0.250**	**0.000**	**0.000**	**0.000**
SD			0.587	0.562	0.000	0.000	
MEAN + SD			**0.937**	**0.812**	**0.000**	**0.000**	**0.000**
			E-PI48	*O-PI48*	*E-PI72*	*O-PI72*	*PIM*

Table S2. Effect of the *C. sinensis* extract (CsEx) on skin energy boosting of treated volunteers after a) 7 b)14 and c) 28 days of treatment. Skin energy value for each volunteer and their variation are reported, along with average percentage variation value ± SD.

a)

SKIN ENERGY D0 - D7<br>CsEx				
**#Volunteer**	**D0**	**D7**	**Δ(D7-D0)**	**Δ(D7-D0)%**
1	3.7	2.9	-0.8	-21.6
2	4.2	4.6	0.4	9.1
3	3.5	4.4	0.9	26.8
4	3.7	3.4	-0.3	-8.4
5	3.5	4.0	0.5	14.1
6	3.5	3.3	-0.2	-4.3
7	3.8	3.8	0.0	0.0
8	3.7	3.8	0.1	3.0
9	3.8	4.0	0.2	5.3
10	3.4	3.7	0.3	7.4
11	3.6	4.7	1.1	30.6
12	3.6	4.6	1.0	27.8
13	3.6	4.6	1.0	27.8
14	3.8	4.1	0.3	7.9
15	3.5	5.0	1.5	42.9
16	3.3	4.0	0.7	21.2
17	3.5	4.9	1.5	42.0
18	3.8	5.1	1.3	34.2
19	4.0	5.3	1.3	32.5
20	3.4	4.8	1.4	41.2
**Average**	3.6	4.2	0.6	**17.0**
**Std. Dev**	0.2	0.7	0.7	18.4
N. Volunteers	20	20	20	
t-test	3.9382494			
Degrees of Freedom	38			
p	0.000339			

b)

SKIN ENERGY D0 - D14<br>CsEx				
**#Volunteer**	**D0**	**D14**	**Δ(D14-D0)**	**Δ(D14-D0)%**
1	3.7	4.2	0.5	12.9
2	4.2	5.2	1.0	22.9
3	3.5	4.6	1.2	33.6
4	3.7	5.0	1.3	34.8
5	3.5	5.4	1.9	55.7
6	3.5	3.7	0.2	6.3
7	3.8	4.2	0.4	10.5
8	3.7	4.1	0.4	10.8
9	3.8	4.7	0.9	24.5
10	3.4	3.8	0.4	12.1
11	3.6	3.8	0.2	5.8
12	3.6	4.2	0.6	16.1
13	3.6	4.6	1.0	27.8
14	3.8	5.0	1.2	31.6
15	3.5	4.70	1.2	34.3
16	3.3	4.45	1.2	34.8
17	3.5	4.90	1.5	42.0
18	3.8	5.10	1.3	34.2
19	4.0	4.70	0.7	17.5
20	3.4	4.80	1.4	41.2
**Average**	3.6	4.6	0.9	**25.5**
**Std. Dev**	0.2	0.5	0.5	13.7
N. Volunteers	20	20	20	
t-test	7.60991843			
Degrees of Freedom	38			
p	3.7349E-09			

c)

SKIN ENERGY D0 - D28<br>CsEx				
**#Volunteer**	**D0**	**D28**	**Δ(D28-D0)**	**Δ(D28-D0)%**
1	3.7	5.1	1.4	37.8
2	4.2	5.2	0.9	22.0
3	3.5	6.3	2.8	81.6
4	3.7	5.3	1.6	42.9
5	3.5	5.5	2.0	58.0
6	3.5	4.5	1.0	29.0
7	3.8	6.8	3.0	78.9
8	3.7	5.1	1.4	38.1
9	3.8	4.5	0.7	18.4
10	3.4	7.4	4.0	118.5
11	3.6	5.2	1.6	43.8
12	3.6	6.3	2.7	75.0
13	3.6	4.6	1.0	27.8
14	3.8	6.0	2.2	57.9
15	3.5	5.3	1.8	51.4
16	3.3	5.0	1.7	51.5
17	3.5	4.3	0.9	24.6
18	3.8	5.0	1.2	31.6
19	4.0	4.9	0.9	22.5
20	3.4	5.2	1.8	52.9
**Average**	3.6	5.4	1.7	**52.0**
**Std. Dev**	0.2	0.8	0.9	25.2
N. Volunteers	20	20	20	
t-test	9.2186724			
Degrees of Freedom	38			
p	3.115E-11			

Table S3. Skin energy on volunteers treated with placebo after a) 7, b) 14, and c) 28 days of treatment. Skin energy value for each volunteer and their variation are reported, along with average percentage variation value ± SD.

a)

SKIN ENERGY D0 - D7 Placebo				
#Volunteer	D0	D7	Δ(D7-D0)	Δ(D7-D0)%
1	3.3	4.9	1.6	47.9
2	3.7	5.7	2.0	53.8
3	3.6	3.8	0.2	5.6
4	3.4	3.5	0.1	4.1
5	3.8	3.4	-0.4	-9.5
6	3.5	3.4	-0.1	-2.6
7	3.4	3.5	0.1	2.9
8	3.8	3.9	0.2	4.0
9	3.6	3.9	0.3	8.9
10	4.0	3.5	-0.5	-12.6
11	3.8	3.9	0.1	3.4
12	3.6	3.5	-0.1	-2.8
13	3.7	3.2	-0.5	-13.5
14	4.0	4.0	0.0	0.0
15	3.5	3.45	0.0	-1.4
16	3.9	3.50	-0.4	-10.3
17	4.0	3.8	-0.2	-5.0
18	3.6	3.7	0.1	2.8
19	3.5	3.4	-0.1	-2.9
20	3.7	3.2	-0.5	-13.5
**Average**	3.7	3.8	0.1	**3.0**
**Std. Dev**	0.2	0.6	0.6	17.7
N. Volunteers	20	20	20	
t-test	0.6872457			
Degrees of Freedom	38			
p	0.4961012			

b)

SKIN ENERGY D0 - D14 Placebo				
#Volunteer	D0	D14	Δ(D14-D0)	Δ(D14-D0)%
1	3.3	4.0	0.7	21.2
2	3.7	4.2	0.5	14.1
3	3.6	4.4	0.8	20.8
4	3.4	4.5	1.1	32.4
5	3.8	5.0	1.2	30.3
6	3.5	4.2	0.7	20.0
7	3.4	3.8	0.4	11.8
8	3.8	4.4	0.6	15.8
9	3.6	4.8	1.2	33.1
10	4.0	4.8	0.8	19.0
11	3.8	3.5	-0.3	-6.8
12	3.6	3.4	-0.2	-5.6
13	3.7	3.6	-0.1	-2.7
14	4.0	4.8	0.8	20.9
15	3.5	4.00	0.5	14.3
16	3.9	4.40	0.5	12.8
17	4.0	5.00	1.0	25.0
18	3.6	4.20	0.6	16.7
19	3.5	4.00	0.5	14.3
20	3.7	4.90	1.2	32.4
**Average**	3.7	4.3	0.6	**17.0**
**Std. Dev**	0.2	0.5	0.4	11.6
N. Volunteers	20	20	20	
t-test	5.26063449			
Degrees of Freedom	38			
p	5.8664E-06			

c)

SKIN ENERGY D0 - D28 Placebo				
#Volunteer	D0	D28	Δ(D28-D0)	Δ(D28-D0)%
1	3.3	4.0	0.7	21.2
2	3.7	6.5	2.8	75.1
3	3.6	4.0	0.4	11.1
4	3.4	5.5	2.1	60.9
5	3.8	3.9	0.1	3.7
6	3.5	4.2	0.7	20.0
7	3.4	4.1	0.7	20.6
8	3.8	5.0	1.2	31.1
9	3.6	4.7	1.1	30.0
10	4.0	3.3	-0.7	-18.0
11	3.8	3.5	-0.3	-6.8
12	3.6	3.6	0.0	0.0
13	3.7	3.6	-0.1	-2.7
14	4.0	4.8	0.8	20.9
15	3.5	4.50	1.0	28.6
16	3.9	4.20	0.3	7.7
17	4.0	4.50	0.5	12.5
18	3.6	4.80	1.2	33.3
19	3.5	4.00	0.5	14.3
20	3.7	3.00	-0.7	-18.9
**Average**	3.7	4.3	0.6	**19.0**
**Std. Dev**	0.2	0.8	0.8	23.2
N. Volunteers	20	20	20	
t-test	3.3381377			
Degrees of Freedom	38			
p	0.0018968			

Table S4. Effect of the *C. sinensis* extract (CsEx) on collagen production in treated volunteers after a) 7 b)14 and c) 28 days of treatment. Collagen Index value for each volunteer and their variation are reported, along with average percentage variation value ± SD.

a)

COLLAGEN INDEX D0 - D7 CsEx				
#Volunteer	D0	D7	Δ(D7-D0)	Δ(D7-D0)%
1	45.4	46.0	0.6	1.3
2	42.1	43.1	1.0	2.4
3	40.4	36.6	-3.8	-9.3
4	41.7	44.6	2.9	7.0
5	39.7	43.1	3.4	8.6
6	40.1	42.8	2.7	6.7
7	38.6	38.6	0.0	0.0
8	41.7	44.7	3.0	7.2
9	41.9	38.9	-3.0	-7.2
10	41.0	38.0	-3.0	-7.4
11	46.1	43.1	-3.1	-6.6
12	45.7	44.6	-1.2	-2.5
13	39.3	39.9	0.6	1.5
14	40.9	42.2	1.3	3.1
15	39.7	43.00	3.3	8.3
16	40.3	42.20	1.9	4.7
17	40.4	42.90	2.5	6.2
18	39.5	41.60	2.1	5.3
19	39.2	41.90	2.7	6.9
20	42.0	39.60	-2.4	-5.7
**Average**	41.3	41.9	0.6	**1.5**
**Std. Dev**	2.2	2.5		
N. Volunteers	20	20	20	
t-test	0.7821607			
Degrees of Freedom	38			
p	0.438967			

b)

COLLAGEN INDEX D0 - D14 Cs Ex				
#Volunteer	D0	D14	Δ(D14-D0)	Δ(D14-D0)%
1	45.4	46.3	0.9	1.9
2	42.1	43.4	1.3	3.1
3	40.4	45.7	5.3	13.1
4	41.7	42.9	1.2	2.9
5	39.7	45.4	5.7	14.2
6	40.1	44.9	4.8	12.1
7	38.6	39.7	1.1	2.8
8	41.7	52.0	10.3	24.7
9	41.9	46.0	4.1	9.8
10	41.0	41.2	0.2	0.6
11	46.1	49.0	2.9	6.2
12	45.7	53.6	7.9	17.2
13	36.3	36.7	0.4	1.1
14	40.9	38.9	-2.0	-5.0
15	39.7	44.8	5.1	12.8
16	40.3	47.6	7.3	18.1
17	40.4	46.9	6.5	16.1
18	39.5	46.8	7.3	18.5
19	39.2	45.1	5.9	15.1
20	42.0	48.0	6.0	14.3
**Average**	41.1	45.2	4.1	**10.0**
**Std. Dev**	2.4	4.1	3.2	7.8
N. Volunteers	20	20	20	
t-test	3.8645388			
Degrees of Freedom	38			
p	0.0004212			

c)

COLLAGEN INDEX D0 - D28<br>CsEx				
**#Volunteer**	**D0**	**D28**	**Δ(D28-D0)**	**Δ(D28-D0)%**
1	45.4	41.3	-4.1	-9.1
2	42.1	31.4	-10.7	-25.4
3	40.4	47.1	6.7	16.6
4	41.7	46.1	4.4	10.6
5	39.7	44.1	4.4	11.1
6	40.1	48.0	7.9	19.6
7	38.6	39.1	0.5	1.3
8	41.7	43.3	1.6	3.8
9	41.9	43.0	1.1	2.7
10	41.0	48.6	7.6	18.6
11	46.1	48.5	2.4	5.2
12	45.7	54.9	9.2	20.1
13	36.3	40.5	4.2	11.6
14	40.9	40.1	-0.8	-1.9
15	39.7	52.0	12.3	31.0
16	40.3	50.1	9.8	24.3
17	40.4	46.2	5.8	14.3
18	39.5	48.2	8.7	21.9
19	39.2	46.2	7.0	17.9
20	42.0	49.0	7.0	16.7
**Average**	41.1	45.4	4.2	**10.5**
**Std. Dev**	2.4	5.2	5.3	12.9
N. Volunteers	20	20	20	
t-test	3.2871185			
Degrees of Freedom	38			
p	0.0021843			

Table S5. Collagen production on volunteers treated with placebo after a) 7, b) 14, and c) 28 days of treatment. Collagen index value for each volunteer and their variation are reported, along with average percentage variation value ± SD.

a)

COLLAGEN INDEX D0 - D7 Placebo				
#Volunteer	D0	D7	Δ(D7-D0)	Δ(D7-D0)%
1	37.7	41.8	4.1	10.9
2	36.9	30.1	-6.8	-18.5
3	40.3	40.9	0.6	1.5
4	44.3	45.70	1.4	3.2
5	39.5	47.0	7.5	19.0
6	37.0	25.6	-11.4	-30.8
7	41.6	30.8	-10.8	-26.0
8	31.4	32.9	1.5	4.8
9	32.2	27.3	-4.9	-15.2
10	40.6	35.5	-5.1	-12.6
11	43.0	42.3	-0.7	-1.6
12	40.7	35.6	-5.1	-12.5
13	41.3	48.3	7.0	16.9
14	47.2	47.8	0.6	1.3
15	39.7	39.90	0.2	0.5
16	41.2	41.40	0.2	0.5
17	40.0	41.50	1.5	3.8
18	41.9	38.50	-3.4	-8.1
19	42.6	43.60	1.0	2.3
20	43.0	43.00	0.0	0.0
**Average**	40.1	39.0	-1.1	**-3.0**
**Std. Dev**	3.7	6.8		
N. Volunteers	20	20	20	
t-test	0.6550035			
Degrees of Freedom	38			
p	0.5164112			

b)

COLLAGEN INDEX D0 - D14 Placebo				
#Volunteer	D0	D14	Δ(D14-D0)	Δ(D14-D0)%
1	37.7	42.5	4.8	12.7
2	36.9	33.7	-3.3	-8.8
3	40.3	45.5	5.2	12.9
4	44.3	48.7	4.4	9.9
5	39.5	43.0	3.5	8.8
6	37.0	34.3	-2.7	-7.3
7	41.6	34.1	-7.5	-18.0
8	31.4	39.5	8.1	25.8
9	32.2	36.2	4.0	12.4
10	40.6	38.2	-2.4	-5.9
11	43.0	49.6	6.6	15.3
12	40.7	33.8	-6.9	-17.0
13	41.3	37.4	-3.9	-9.4
14	47.2	49.5	2.3	4.9
15	39.7	42.8	3.1	7.8
16	41.2	42.9	1.7	4.1
17	40.0	41.9	1.9	4.8
18	41.9	43.8	1.9	4.5
19	42.6	45.1	2.5	5.9
20	43.0	45.6	2.6	6.0
**Average**	40.1	41.4	1.3	**3.5**
**Std. Dev**	3.7	5.3	4.3	11.2
N. Volunteers	20	20	20	
t-test	0.8986201			
Degrees of Freedom	38			
p	0.374514			

c)

COLLAGEN INDEX D0 - D28<br>Placebo				
**#Volunteer**	**D0**	**D28**	**Δ(D28-D0)**	**Δ(D28-D0)%**
1	37.7	44.1	6.4	17.0
2	36.9	35.4	-1.5	-4.0
3	40.3	40.9	0.6	1.5
4	44.3	36.9	-7.4	-16.7
5	39.5	41.6	2.1	5.4
6	37.0	28.6	-8.4	-22.7
7	41.6	30.7	-10.9	-26.2
8	31.4	31.7	0.3	1.0
9	32.2	30.9	-1.3	-4.0
10	40.6	29.5	-11.1	-27.3
11	43.0	46.7	3.7	8.6
12	40.7	32.6	-8.1	-19.9
13	41.3	37.0	-4.3	-10.4
14	47.2	31.4	-15.8	-33.5
15	39.7	39.50	-0.2	-0.5
16	41.2	37.80	-3.4	-8.3
17	40.0	34.40	-5.6	-14.0
18	41.9	38.50	-3.4	-8.1
19	42.6	39.30	-3.3	-7.7
20	43.0	38.40	-4.6	-10.7
**Average**	40.1	36.3	-3.8	**-9.0**
**Std. Dev**	3.7	5.0	5.4	12.9
N. Volunteers	20	20	20	
t-test	2.7215356			
Degrees of Freedom	38			
p	0.0097516			

Table S6. T-zone wrinkles on volunteers treated with CsEx after a) 7, b) 14, and c) 28 days. T-zone wrinkles value for each volunteer and their variation are reported, along with average percentage variation value ± SD.

a)

**T-ZONE WRINKLES D0 - D7** CsEx				
**#Volunteer**	**D0**	**D7**	**Δ(D7-D0)**	**Δ(D7-D0)%**
1	20.50	21.67	1.2	5.7
2	37.58	23.98	-13.6	-36.2
3	24.19	20.95	-3.2	-13.4
4	25.05	23.57	-1.5	-5.9
5	32.32	32.74	0.4	1.3
6	20.34	14.03	-6.3	-31.0
7	29.56	19.50	-10.1	-34.1
8	20.71	16.15	-4.6	-22.0
9	26.00	21.69	-4.3	-16.6
10	33.47	21.26	-12.2	-36.5
11	26.65	19.03	-7.6	-28.6
12	22.60	18.39	-4.2	-18.7
13	26.48	22.40	-4.1	-15.4
14	24.06	25.77	1.7	7.1
15	24.55	20.78	-3.8	-15.4
16	29.60	27.55	-2.1	-6.9
17	21.70	23.70	2.0	9.2
18	26.80	27.40	0.6	2.2
19	27.20	28.00	0.8	2.9
20	26.45	27.00	0.6	2.1
**Average**	26.29	22.78	-3.5	**-12.5**
**Std. Dev**	4.5	4.5	4.6	15.4
N. Volunteers	20	20	20	
t-test	2.4771067			
Degrees of Freedom	38			
p	0.017809			

b)

**T-ZONE WRINKLES D0 - D14** CsEx				
**#Volunteer**	**D0**	**D14**	**Δ(D14-D0)**	**Δ(D14-D0)%**
1	20.50	20.56	0.1	0.3
2	37.58	24.23	-13.4	-35.5
3	24.19	17.78	-6.4	-26.5
4	25.05	24.10	-0.9	-3.8
5	32.32	19.46	-12.9	-39.8
6	20.34	13.49	-6.8	-33.7
7	29.56	27.13	-2.4	-8.2
8	20.71	15.04	-5.7	-27.4
9	26.00	25.01	-1.0	-3.8
10	33.47	25.15	-8.3	-24.9
11	26.65	19.19	-7.5	-28.0
12	22.60	19.75	-2.9	-12.6
13	26.48	24.51	-2.0	-7.5
14	24.06	14.51	-9.5	-39.7
15	24.55	20.35	-4.2	-17.1
16	29.60	23.55	-6.1	-20.4
17	21.70	22.75	1.1	4.8
18	26.80	17.40	-9.4	-35.1
19	27.20	24.39	-2.8	-10.3
20	26.45	23.50	-3.0	-11.2
**Average**	26.29	21.09	-5.2	**-19.0**
**Std. Dev**	4.5	3.9	4.1	13.9
N. Volunteers	20	20	20	
t-test	3.878287			
Degrees of Freedom	38			
p	0.000405			

c)

T-ZONE WRINKLES D0 - D28<br>CsEx				
**#Volunteer**	**D0**	**D28**	**Δ(D28-D0)**	**Δ(D28-D0)%**
1	20.50	13.40	-7.1	-34.6
2	37.58	24.08	-13.5	-35.9
3	24.19	14.22	-10.0	-41.2
4	25.05	23.15	-1.9	-7.6
5	32.32	24.59	-7.7	-23.9
6	20.34	14.03	-6.3	-31.0
7	29.56	15.85	-13.7	-46.4
8	20.71	14.99	-5.7	-27.6
9	26.00	19.38	-6.6	-25.5
10	33.47	19.59	-13.9	-41.5
11	26.65	11.37	-15.3	-57.4
12	22.60	21.22	-1.4	-6.1
13	26.48	24.62	-1.9	-7.0
14	24.06	6.48	-17.6	-73.1
15	24.55	23.10	-1.5	-5.9
16	29.60	30.00	0.4	1.4
17	21.70	17.00	-4.7	-21.7
18	26.80	19.35	-7.5	-27.8
19	27.20	21.60	-5.6	-20.6
20	26.45	19.50	-7.0	-26.3
**Average**	26.29	18.88	-7.4	**-28.0**
**Std. Dev**	4.5	5.5	5.1	18.5
N. Volunteers	20	20	20	
t-test	4.6482833			
Degrees of Freedom	38			
p	3.958E-05			

Table S7. T-zone wrinkles on volunteers treated with placebo after a) 7, b) 14, and c) 28 days. T-zone wrinkles value for each volunteer and their variation are reported, along with average percentage variation value ± SD.

a)

**T-ZONE WRINKLES D0 - D7** Placebo				
**#Volunteer**	**D0**	**D7**	**Δ(D7-D0)**	**Δ(D7-D0)%**
1	32.02	39.06	7.0	22.0
2	19.34	11.56	-7.8	-40.2
3	31.00	34.60	3.6	11.6
4	34.63	32.69	-1.9	-5.6
5	36.86	34.49	-2.4	-6.4
6	29.68	22.40	-7.3	-24.5
7	32.81	27.33	-5.5	-16.7
8	28.57	24.79	-3.8	-13.3
9	33.93	33.46	-0.5	-1.4
10	34.78	39.69	4.9	14.1
11	28.98	49.78	20.8	71.8
12	31.41	55.78	24.4	77.6
13	30.00	30.00	0.0	0.0
14	33.74	71.74	38.0	112.6
15	28.80	32.80	4.0	13.9
16	30.55	30.95	0.4	1.3
17	27.60	30.55	3.0	10.7
18	26.80	30.80	4.0	14.9
19	32.25	40.60	8.4	25.9
20	30.95	37.77	6.8	22.0
**Average**	30.74	35.54	4.8	**14.5**
**Std. Dev**	3.7	12.6	11.3	36.2
N. Volunteers	20	20	20	
t-test	1.6308293			
Degrees of Freedom	38			
p	0.1111883			

b)

**T-ZONE WRINKLES D0 - D14** Placebo				
**#Volunteer**	**D0**	**D14**	**Δ(D14-D0)**	**Δ(D14-D0)%**
1	32.02	36.60	4.6	14.3
2	19.34	18.40	-0.9	-4.8
3	31.00	28.70	-2.3	-7.4
4	34.63	30.09	-4.5	-13.1
5	36.86	38.98	2.1	5.7
6	29.68	23.36	-6.3	-21.3
7	32.81	25.86	-7.0	-21.2
8	28.57	24.91	-3.7	-12.8
9	33.93	38.85	4.9	14.5
10	34.78	30.56	-4.2	-12.1
11	28.98	26.47	-2.5	-8.6
12	31.41	31.42	0.0	0.0
13	40.00	40.00	0.0	0.0
14	33.74	30.03	-3.7	-11.0
15	28.80	24.35	-4.5	-15.5
16	30.55	26.80	-3.8	-12.3
17	27.60	30.70	3.1	11.2
18	36.80	42.78	6.0	16.3
19	32.25	29.55	-2.7	-8.4
20	30.95	26.70	-4.3	-13.7
**Average**	31.74	30.25	-1.5	**-5.0**
**Std. Dev**	4.3	6.3	3.8	11.8
N. Volunteers	20	20	20	
t-test	0.866672			
Degrees of Freedom	38			
p	0.391561			

c)

T-ZONE WRINKLES D0 - D28<br>NC69 - CORDYS REVITAGEN<br>Placebo				
**#Volunteer**	**D0**	**D28**	**Δ(D28-D0)**	**Δ(D28-D0)%**
1	32.02	36.59	4.6	14.3
2	19.34	10.62	-8.7	-45.1
3	31.00	28.00	-3.0	-9.7
4	34.63	35.21	0.6	1.7
5	36.86	58.40	21.5	58.4
6	29.68	17.36	-12.3	-41.5
7	32.81	21.55	-11.3	-34.3
8	28.57	13.54	-15.0	-52.6
9	33.93	38.28	4.3	12.8
10	34.78	25.78	-9.0	-25.9
11	28.98	29.32	0.3	1.2
12	31.41	42.90	11.5	36.6
13	40.00	40.00	0.0	0.0
14	33.74	64.00	30.3	89.7
15	28.80	31.00	2.2	7.6
16	30.55	32.00	1.5	4.7
17	27.60	27.20	-0.4	-1.4
18	36.80	36.00	-0.8	-2.2
19	32.25	35.50	3.3	10.1
20	30.95	32.50	1.6	5.0
**Average**	31.74	32.79	1.1	**1.5**
**Std. Dev**	4.3	13.0	10.8	33.9
N. Volunteers	20	20	20	
t-test	0.34421241			
Degrees of Freedom	38			
p	0.73258417			

Table S8. Anova test on CsEx skin energy value vs placebo at D7, D14, and D28.

	SKIN ENERGY D7		SKIN ENERGY D14		SKIN ENERGY D28	
	CsEx	Placebo	CsEx	Placebo	CsEx	Placebo
	-21.62	47.88	12.89	21.21	37.84	21.21
	9.13	53.80	22.93	14.13	21.99	75.14
	26.80	5.56	33.57	20.83	81.56	11.11
	-8.36	4.09	34.77	32.35	42.86	60.88
	14.08	-9.47	55.75	30.26	58.05	3.68
	-4.31	-2.58	6.32	20.00	29.02	20.00
	0.00	2.94	10.53	11.76	78.95	20.59
	3.00	4.00	10.81	15.79	38.08	31.05
	5.26	8.89	24.47	33.06	18.42	30.00
	7.35	-12.60	12.06	19.00	118.50	-18.00
	30.56	3.42	5.83	-6.80	43.78	-6.84
	27.78	-2.78	16.11	-5.56	75.00	0.00
	27.78	-13.51	27.78	-2.70	27.78	-2.70
	7.89	0.00	31.58	20.90	57.89	20.90
	42.86	-1.43	34.29	14.29	51.43	28.57
	21.21	-10.26	34.85	12.82	51.52	7.69
	42.03	-5.00	42.03	25.00	24.64	12.50
	34.21	2.78	34.21	16.67	31.58	33.33
	32.50	-2.86	17.50	14.29	22.50	14.29
	41.18	-13.51	41.18	32.43	52.94	-18.92
Group average	16.966317	2.96782061	25.47288307	16.98672529	48.21536401	17.22424274
Total average	9.967068803		21.22980418		32.71980338	
Number of data	20		20		20	
SSB	1959.579011		720.1487398		9604.495977	
SSx	6403.788352	5950.292432	3561.556509	2565.019462	12061.78105	10213.37969
SSW	12354.08078		6126.575971		22275.16074	
dfB	1		1		1	
dfS	38		38		38	
S^2B	1959.579011		720.1487398		9604.495977	
S^2W	325.1073891		161.2256834		586.1884405	
F	6.027482231		4.466712278		16.38465605	
Significance level	0.05		0.05		0.05	
pvalue	0.018774072		0.041184105		0.000244989	
	$		$		$$$	

Table S9. Anova test on CsEx collagen index value vs placebo at D7, D14, and D28.

	COLLAGEN INDEX D7		COLLAGEN INDEX D14		COLLAGEN INDEX D28	
	CsEx	Placebo	CsEx	Placebo	CsEx	Placebo
	1.32	10.88	1.89	12.73	-9.10	16.98
	2.38	-18.51	3.09	-8.81	-25.42	-4.00
	-9.31	1.49	13.09	12.90	16.61	1.49
	6.95	3.16	2.88	9.93	10.55	-16.70
	8.56	18.99	14.23	8.80	11.08	5.40
	6.71	-30.81	12.06	-7.30	19.58	-22.70
	0.00	-25.96	2.85	-18.03	1.30	-26.20
	7.19	4.78	24.68	25.80	3.79	0.96
	-7.16	-15.22	9.79	12.42	2.70	-4.04
	-7.41	-12.56	0.60	-5.91	18.56	-27.34
	-6.62	-1.63	6.18	15.35	5.23	8.60
	-2.52	-12.53	17.20	-16.95	20.09	-19.90
	1.53	16.95	1.10	-9.44	11.57	-10.41
	3.15	1.27	-5.00	4.87	-1.90	-33.47
	8.31	0.50	12.85	7.81	30.98	-0.50
	4.71	0.49	18.11	4.13	24.32	-8.25
	6.19	3.75	16.09	4.75	14.28	-14.00
	5.32	-8.11	18.48	4.53	21.90	-8.11
	6.89	2.35	15.05	5.87	17.86	-7.75
	-5.71	0.00	14.29	6.05	16.67	-10.70
Group average	1.524249	-3.036907996	9.97549074	3.475041041	10.53164405	-9.033194638
Total average	-0.756329481		6.72526589		0.749224707	
Number of data	20		20		20	
SSB	208.0415346		422.5584629		3827.82913	
SSx	672.5767283	3219.097792	1148.258876	2401.780037	3171.317918	3152.980914
SSW	3891.67452		3550.038913		6324.298832	
dfB	1		1		1	
dfS	38		38		38	
S^2B	208.0415346		422.5584629		3827.82913	
S^2W	102.4124874		93.42207666		166.4289166	
F	2.031407887		4.523111432		22.99978398	
Significance level	0.05		0.05		0.05	
pvalue	0.162240463		0.039992326		2.50723E-05	
			$		$$$	

Table S10. Anova test on CsEx T-zone wrinkles value vs placebo at D7, D14, and D28.

	T-ZONE WRINKLES D7		T-ZONEWRINKLES D14		T-ZONE WRINKLES D28	
	CsEx	Placebo	CsEx	Placebo	CsEx	Placebo
	5.68	21.99	0.30	14.30	-34.62	14.27
	-36.46	-40.20	-35.52	-4.84	-35.93	-45.07
	-15.88	11.61	-26.51	-7.42	-41.22	-9.68
	-7.90	-5.60	-3.78	-13.11	-7.59	1.68
	1.30	-6.44	-39.81	5.75	-23.93	58.45
	-31.03	-24.54	-33.66	-21.32	-31.03	-41.51
	-34.06	-16.70	-8.23	-21.20	-46.40	-34.32
	-22.00	-13.26	-27.40	-12.83	-27.61	-52.62
	-16.56	-1.40	-3.81	14.49	-25.47	12.80
	-36.48	14.12	-24.86	-12.15	-41.47	-25.89
	-28.61	71.80	-28.00	-8.63	-57.35	1.19
	-18.66	77.58	-12.62	0.05	-6.10	36.57
	-15.42	0.00	-7.45	0.00	-7.03	0.00
	7.12	112.60	-39.67	-11.00	-73.07	89.69
	-15.36	13.89	-17.11	-15.45	-5.91	7.64
	-6.93	1.31	-20.44	-12.27	1.35	4.75
	9.22	10.69	4.84	11.23	-21.66	-1.45
	2.24	14.93	-35.07	16.25	-27.05	-2.17
	3.49	25.89	-10.33	-8.37	-19.85	10.08
	2.46	22.04	-11.15	-13.73	-25.90	5.01
**Group average**	-12.690967	14.51403166	-19.01460344	-5.013389202	-27.89157271	1.470117335
**Total average**	0.911532556		-12.01399632		-13.21072769	
**Number of data**	20		20		20	
**SSB**	7401.11927		1960.340001		8621.088421	
**SSx**	4514.437736	24892.82987	3684.54762	2644.899453	6523.881252	21804.8603
**SSW**	29407.26761		6329.447073		28328.74155	
**dfB**	1		1		1	
**dfS**	38		38		38	
**S^2B**	7401.11927		1960.340001		8621.088421	
**S^2W**	773.8754633		166.5643967		745.4931987	
**F**	9.563708401		11.76926186		11.56427508	
**Significance level**	0.05		0.05		0.05	
**pvalue**	0.003709136		0.001465214		0.001593728	
	$$		$$		$$	
